# Teachers’ mental health from the perspectives of moral injury and institutional betrayal, and on trauma-sensitive organizational environments: a narrative review

**DOI:** 10.12701/jyms.2026.43.57

**Published:** 2026-08-18

**Authors:** So Hye Jo

**Affiliations:** Department of Psychiatry, Yeungnam University College of Medicine, Daegu, Korea

**Keywords:** Employee assistance programs, Mental health, Moral injury, Occupational stress, School teachers

## Abstract

Teachers’ mental health is a critical public health concern in South Korea. The current educational environment, characterized by intensifying parent-teacher conflicts, legal disputes, and increasing administrative burdens, demonstrates the limitations of interpreting these challenges solely through the lens of individual vulnerability or resilience. Moral injury can provide a useful explanatory framework for understanding how chronic occupational stressors and institutional betrayal lead to negative mental health outcomes among teachers. In this narrative literature review, I examine new concepts related to teachers’ psychological distress, including moral injury, institutional betrayal, secondary trauma, and occupational mental health. With a particular focus on preventive and trauma-informed approaches, I also review international employee assistance program (EAP) models and organizational support systems. International case studies emphasize the importance of confidential EAP services, multidisciplinary support systems, administrative support, and organizational resources to enhance the psychological safety of teachers and promote early intervention. Based on the reviewed literature, this study proposes the development of an independent EAP model for Korean teachers and an institutional support system that reflects an understanding of trauma, with the aim of enhancing teachers’ well-being and contributing to the creation of a sustainable education system.

## Introduction

Protecting the authority and educational activities of teachers has emerged as a major social concern in South Korea [1]. South Korea’s public education system is currently facing a complex crisis characterized by the erosion of teacher authority and psychological burnout. Recently, as supervising teachers have been held criminally and civilly liable for accidents that occur during extracurricular activities, teachers’ concerns about unpredictable legal risks have grown. Furthermore, legitimate guidance and educational activities by teachers are increasingly becoming the subject of child abuse reports or parental complaints, further exacerbating the psychological burden on teachers.

These factors affect teachers’ mental health and professional identity, leading to depression, anxiety, post-traumatic stress symptoms, emotional exhaustion, and increased intention to leave the teaching profession. Although teachers are dedicated to protecting and educating students, they are often unable to fully realize their mission, leading them to repeatedly experience feelings of helplessness, guilt, anger, and betrayal. These mental health issues among teachers should be understood as a result of the interplay between moral injury caused by the erosion of professional values, institutional betrayal resulting from a lack of organizational protection, and trauma arising from repeated exposure to crises involving students and schools. However, efforts to examine the nature of teachers’ psychological distress fully have been limited. Therefore, to better understand and address mental health crises in the teaching profession, a psychiatric understanding of the environmental and emotional conditions underlying teachers’ distress is essential.

In response to these issues, this review reexamines the current collective mental health crisis among Korean teachers from the perspectives of moral trauma and organizational structure. It also examines international teacher support systems and occupational mental health models to identify, from the perspectives of psychiatry and public health, the teacher mental health support system needed in the Korean educational context and the direction for developing an employee assistance program (EAP) tailored to South Korea. Accordingly, this review proposes a conceptual framework in which changes in social and educational environments expose teachers to potentially morally injurious events that contribute to moral injury, institutional betrayal, and subsequent psychiatric and occupational outcomes (Fig. 1).

## Literature search and selection

This narrative review was informed by the literature identified through searches of PubMed, Google Scholar, Regional Information Sharing Service (RISS), and DBpia, supplemented by relevant governmental, professional, and institutional sources. The search included literature available through June 2026 and used a combination of terms related to “teacher mental health,” “teacher burnout,” “moral injury,” “institutional betrayal,” “secondary traumatic stress,” “occupational stress,” “psychological safety,” “employee assistance program,” “teacher support,” and “school organizational support.” Peer-reviewed empirical studies, systematic and narrative reviews, and theoretical papers addressing teacher mental health, moral injury, institutional betrayal, occupational stress, and trauma-related psychological outcomes were prioritized. Major governmental, professional, and policy documents relevant to educational policies, occupational mental health, crisis intervention, and organizational support systems were also included. International examples from the United States, the United Kingdom, and Finland were selected to illustrate distinct organizational approaches, including tiered and multidisciplinary student support, EAPs, occupational stress management, and school-based welfare systems, rather than to provide a systematic cross-national comparison.

## Mental health crisis among Korean teachers and sociocultural change

The self-discrepancy theory provides a useful psychological framework for understanding how occupational stressors may become morally and emotionally distressing. Teachers often enter the profession with strong internalized ideals of care, protection, fairness, and educational responsibility. When institutional constraints, legal threats, parent–teacher conflicts, or inadequate organizational support prevent teachers from acting in accordance with these values, the resulting discrepancy between professional ideals and actual work experiences may lead to guilt, shame, anger, helplessness, and demoralization [2]. Therefore, understanding the psychological burnout and mental health crises currently evident in the teaching profession requires examining not only the current work environment but also who chooses to become a teacher, the sociocultural context of that choice, and the changes experienced throughout the teaching career.

### 1. Changing reality of the teaching profession, contrary to expectations

As reflected in the saying, “Do not even step on your teacher’s shadow,” teachers have long been held in high esteem in Korean society and are associated with authority. In traditional Confucian culture, based on the principle that “the ruler, the teacher, and the father are one” (君師父一體), teachers were regarded as figures of respect comparable to parents and the state, and served as intellectuals and moral mentors in their communities. For generations raised in this cultural environment, teaching represented more than just a job; it was a profession that enabled social contributions and ethical fulfillment. After employment instability spread following the 1997 International Monetary Fund foreign exchange crisis, teaching became a highly sought-after career for the younger generation because of its stable employment structure and welfare benefits. Many of these students entered the teaching profession after intense competition, driven by professional pride, expectations of social respect, and hope for a stable future.

However, the reality of educational practices often differs significantly from these expectations. As school education shifted toward being treated as a public service and educational consumerism emerged, complaints and legal disputes became recurring issues. Furthermore, as teachers’ administrative responsibilities increased and their emotional labor intensified, they experienced a fundamental shift in their work lives. In addition, teachers are increasingly exposed to relationship-based trauma through student self-harm, suicide risks, conflicts with parents, public complaints, and criticism. Despite their strong sense of professional calling, teachers repeatedly experienced a sense of helplessness in this challenging environment.

In this process, many teachers experience a psychological disconnect between their expected identities as educational professionals or facilitators of student growth and the emotionally demanding roles they encounter in practice [3].

### 2. Indicators of teachers’ mental health

Teachers inevitably engage in prolonged interactions with students. Under these conditions, recurring difficulties and conflicts can affect the professional identity of teachers [4]. This phenomenon is readily evident in several recent surveys on teachers’ occupational and mental health as well as in administrative indicators. According to the 2024 Organisation for Economic Co-operation and Development (OECD) Teaching and Learning International Survey (TALIS), Korean teachers reported lower job satisfaction than the OECD average and had the highest proportion of teachers among participating countries who regretted becoming teachers [5].

According to data submitted to the National Assembly’s Education Committee, the number of new teachers with less than 5 years of experience who left the profession increased by approximately 31% from 290 in 2020 to 380 in 2024 [6]. The fact that approximately 95% of these resignations in 2024 were classified as voluntary raises concerns about structural issues within the teaching profession [7]. According to the 2023 “Survey on teachers’ job-related mental health” conducted by the Korean Teachers and Education Workers Union and Green Hospital, 38.3% of the teachers who participated in the survey exhibited severe symptoms of depression, and 16.0% reported experiencing suicidal thoughts during the past year [8]. These rates were significantly higher than the prevalence of depression and suicidal thoughts observed in the general adult population. Furthermore, a significant number of teachers who had experienced verbal abuse, physical violence, or sexual harassment were identified as being at high risk for post-traumatic stress disorder (PTSD), indicating that repeated complaints and exposure to violence may be closely linked to the deterioration of educators’ mental health.

## Limitations of existing theoretical frameworks for explaining teachers’ mental health

### 1. Limitations of burnout and the job demands-resources model

Burnout is defined by the World Health Organization as an occupational phenomenon resulting from chronic workplace stress that has not been successfully managed, and is characterized by exhaustion, increased mental distance or cynicism toward one’s job, and reduced professional efficacy [9]. Traditionally, health and medical communities, as well as the education sector, have primarily used the concept of “burnout” to explain teachers’ job abandonment and mental health issues. Burnout is primarily associated with excessive workloads and emotional labor. However, psychological distress recently reported within the teaching profession has complex characteristics that are difficult to explain solely by excessive workload or job stress; in some cases, symptoms resembling moral injury and institutional betrayal have been observed. Teacher distress often involves moral conflict, professional identity, educational vocation, and damage to self-concept.

According to the job demands-resources (JD-R) model, when employees are faced with excessive job demands, they experience psychological and physical energy depletion, and their health may deteriorate when they lack accessible job resources [10]. Compared to the burnout model, the JD-R model provides a far more useful approach for understanding structural stressors in the teaching profession, such as increased administrative workload, excessive handling of public complaints, and role overload, which affect the mental health of teachers. However, this model also focuses primarily on the quantitative and external aspects of the workload and work environment. Consequently, it has limitations in fully explaining the erosion of unique professional values such as teachers’ moral and ethical identities or their sense of educational vocation. This is because teaching is a profession deeply connected to a moral purpose, one that goes beyond wage labor and aims for student growth and the realization of social values [11].

This moral commitment is considered a core element of the professional identity of teachers. Therefore, when teachers perceive that they are unable to fulfill a role consistent with their educational beliefs and values, they may experience psychological distress that goes beyond job stress [12]. When teachers are unable to carry out legitimate educational activities, they experience conflict between their obligation to protect students and the need to avoid legal or administrative risks, or perceive that their organization fails to protect them; this distress manifests as damage to their value systems and professional identities. Such experiences may be accompanied by shame, guilt, betrayal, helplessness, and loss of professional meaning; these are better understood through the concepts of moral injury, demoralization, and institutional betrayal rather than burnout alone. Therefore, explaining teachers’ mental health issues requires an expanded mental health model that considers the moral conflicts that teachers experience and their damaged trust in organizations.

### 2. Moral injury and institutional betrayal

#### 1) Moral injury and demoralization

Moral injury refers to the psychological and emotional distress that occurs when an individual experiences or witnesses an event that seriously violates or undermines their internalized moral beliefs or ethical values [13]. Demoralization can occur when teachers fail to receive ongoing moral rewards (such as making a positive contribution to society by guiding students properly and as expected), which are central to their professional sense of meaning and sense of accomplishment [14]. Excessive administrative procedures, rigid systems, and the possibility of recurring complaints and legal disputes can undermine teachers’ sense of professional meaning. Furthermore, these issues can lead to psychological distress such as feelings of helplessness or cynicism toward teaching. If this occurs repeatedly, teachers may blame themselves for inadequacy or lose their faith in education. This is closer to the phenomenology of moral injury, centered on shame, betrayal, and identity collapse, than to simple burnout [15]. Teachers’ moral injury and low morale are primarily related to situations where they experience a disconnect between their own beliefs and reality as agents of change, as well as to vicarious trauma and secondary post-traumatic stress, as explained below [16].

##### (1) Suffering as an act of commission

Suffering as an act of commission refers to suffering that arises from the disconnect between ethical ideals and reality when an individual is compelled to direct or carry out actions that are morally unacceptable to them [13]. Recently, teachers have been faced with the possibility of legal liability owing to safety incidents or complaints arising from their student guidance activities. This forces teachers to prioritize risk minimization over educational values, ultimately leading to overly conservative or passive teaching methods [17]. This trend is confirmed by research showing that teachers tend to limit student participation or scale back creative and autonomous activities to avoid complaints or conflicts. In the long term, such conflicts are likely to have a negative impact on teachers’ moral identities and their sense of professional meaning [14].

##### (2) Pain of being a bystander

The deepening emotional and behavioral problems among adolescents, their risk of suicide, and their violent words and actions place a significant emotional burden on teachers [18]. However, teachers may find it difficult to actively intervene due to institutional constraints and concerns that they might become embroiled in legal disputes, such as allegations of child abuse. In such situations, teachers may feel that they have not responded adequately, leading to ethical conflicts and a sense of helplessness [13]. In the worst situation, when a student experiencing severe emotional crisis or suicidal impulses dies despite the teacher’s efforts, some teachers experience survivor’s guilt and deep self-blame [19]. Such experiences can affect a teacher’s self-image and professional identity, and in the long term, may manifest as post-traumatic stress, depression, anxiety, and avoidance behaviors, leading to difficulties in performing daily and occupational functions [20]. Recent studies have shown that the more teachers are exposed to events that could potentially cause moral harm, the higher their levels of depression, anxiety, burnout, and intention to leave the teaching profession; in particular, shame and guilt have been found to act as key psychological mechanisms mediating these relationships [21].

##### (3) Institutional betrayal

Institutional betrayal refers to the psychological harm experienced when an organization or institution from which an individual expects safety and protection fails to adequately protect its members, downplays or ignores harm, or shifts responsibility onto the individual [22]. This framework posits that traumatic responses intensify when a trusted entity inflicts harm or abdicates its protective role [23].

Teachers work with the fundamental trust that they will be protected and supported within the school system when faced with conflicts or crises in the classroom. However, when schools and school districts passively respond to crises, fail to properly acknowledge the trauma experienced, or focus on shifting blame rather than resolving the problem, the psychological distress of teachers can worsen. Such experiences of institutional betrayal can erode trust in the organization and lead to chronic emotional distress; if these situations recur, they may manifest in patterns similar to complex trauma [22,24].

When faced with prolonged exposure to psychological conflict and trauma while feeling inadequately protected by the organization, a teacher’s long-held professional identity may be fundamentally shaken [25]. At this stage, some teachers may experience major depressive disorder, interpersonal avoidance, complex PTSD symptoms, and post-traumatic resentment accompanied by persistent feelings of injustice and helplessness, which can ultimately increase the risk of leaving the teaching profession or retiring early [26,27].

## Occupational and institutional conditions relevant to teacher distress and moral injury

To better understand the mental health issues and establish practical support systems for teachers, an integrated perspective is needed that considers not only individual-level psychological interventions but also the work environment, organizational structure, and environmental protective factors that foster psychological safety. Importantly, occupational and institutional stressors should not be equated to moral injury. Rather, they may contribute to moral injury when they create persistent conflicts with teachers’ professional values, constrain ethically appropriate actions, or are accompanied by perceived institutional betrayal.

### 1. Structure of exposure to parent complaints

In South Korea, measures such as recording systems for school landline phones, manuals for handling parent complaints, and dedicated complaint interview rooms have recently been introduced. However, in actual school settings, individual teachers are still often required to handle complaints directly [28]. Some teachers are even contacted outside of working hours via their personal phone numbers or mobile messaging apps or have to deal directly with parents who visit unannounced. This repeated exposure causes high levels of stress and anticipatory anxiety owing to its unpredictable nature. In cases involving high-intensity complaints or potential legal disputes, a structural approach is needed, in which mediation systems at the school administration or local education office level intervene to reduce the direct exposure of individual teachers to repeated conflict.

### 2. Limitations of student support systems

In Korea and internationally, adolescent mental health issues such as depression, anxiety, conduct problems, self-harm, and suicide are increasing annually [29]. Some teachers experience stress because as part of their efforts to address these difficulties in students, including the process of removal, they may face potential conflicts with uncooperative parents or may be implicated in allegations of emotional abuse. Although some legal grounds for student removal have recently been established [30], their effectiveness remains limited due to a lack of separate alternative spaces and human resources capable of professionally intervening in behavioral issues.

Because adolescents spend a significant portion of their day at school, support systems for these students are closely linked to teachers’ mental health, and individual teachers often experience significant psychological strain when working with them [31,32]. Even when it is determined that a student exhibiting problem behavior requires professional treatment, school-level interventions may face limitations owing to a lack of parental consent, insufficient community mental health resources, and restrictions on service access [33]. This can cause teachers to feel helpless, reducing their sense of job efficacy [34].

### 3. Excessive workload and unclear job boundaries

Ambiguity regarding the scope of roles and the boundaries of responsibilities is a major factor affecting teachers’ mental health. According to the OECD TALIS, Korean teachers spend more time on administrative tasks than the OECD average [35]. In addition to teaching and learning activities, teachers expend a great deal of energy in performing various administrative and coordination tasks, such as processing official documents, managing budgets, overseeing facilities, coordinating student care, and responding to public complaints. This can also pose a significant obstacle to maintaining their identity as educators.

In South Korea, “Neulbom Schools,” which focus on after-school care, are in operation. In principle, homeroom teachers are not responsible for after-school care. However, rather than outsourcing the entire operation to private agencies, South Korea operates the program by establishing a new, independent after-school care organization within schools [36]. Although a separate department called the “Neulbom Support Office” exists within schools and is staffed by dedicated personnel, reports indicate that, in practice, part of the workload is being shifted onto teachers due to regional disparities in staffing and administrative gaps [28]. Furthermore, since many parents believe that the school is responsible for after-school care programs, teachers often experience significant psychological stress when complaints or incidents related to after-school care arise.

## Models of the employee assistance program for teachers

Some countries are addressing teachers’ mental health issues not only through individual counseling or depression treatment, but also within an organizational-level, preventive occupational mental health system. In particular, the emphasis is shifting toward EAPs and school-based multidisciplinary support systems designed to enhance teachers’ psychological safety, mitigate job-related stress, reduce exposure to trauma, and establish organizational protection systems. This approach aligns with the broader effort to understand teachers’ mental health issues, not merely as individual vulnerabilities but also as problems rooted in the work environment and organizational structure.

In this review, the United States, the United Kingdom, and Finland were selected as illustrative cases because they represent distinct organizational approaches relevant to teachers’ mental health and occupational support. These countries were not selected to imply that their educational or healthcare systems are directly comparable to those of South Korea or to provide a comprehensive cross-national comparison. Rather, they were purposively selected to illustrate complementary structural approaches: the United States demonstrates tiered student support and externally provides employee assistance services, the United Kingdom emphasizes organization-level management of work-related stress and occupational mental health, and Finland provides an example of preventive, school-based multidisciplinary welfare support. Examining these contrasting models may help to identify organizational principles that could inform the development of teacher mental health support systems in the Korean context (Table 1).

### 1. Multitiered system of support and external employee assistance program in the United States

In the United States public education system, students’ emotional and behavioral issues are widely managed through a multitiered system of supports (MTSS), rather than being handled solely by individual teachers [37]. The MTSS provides tiered support based on the severity of students’ needs. In the early stages, interventions are conducted in the general classroom with counseling support; for high-risk students, a multidisciplinary team comprising school psychologists, social workers, behavior analysts, and special education teachers intervenes [38]. A key feature of this system is that the responsibility for supporting students with behavioral issues is distributed across the school organization rather than concentrated on individual teachers [39].

In addition, some schools operate separate spaces such as “calm-down spaces” or “student support rooms,” for students who have difficulty managing their behavior during class. In certain cases, behavior specialists or student support staff intervene in this process [40]. This approach can be understood as an effort to manage student behavioral problems within a structured behavioral support system rather than as a failure of individual teachers to maintain control.

The United States widely utilizes Positive Behavioral Interventions and Supports (PBIS) as a school-based behavioral intervention model [37,41]. PBIS manages students’ behavioral challenges based on a set of tiered intervention principles that are consistently shared across the entire school. Student behavior is managed through a tiered approach consisting of universal, selective, and intensive individual support. This model is designed such that behavioral interventions are implemented according to standardized principles and procedures rather than relying on the responses of individual teachers, thereby partially alleviating the emotional burden and role conflicts experienced by teachers.

In addition, to protect teachers’ mental health, the United States uses the EAP model, which combines internal counseling with outsourcing management to external, independent agencies [42]. Using outsourced EAPs ensures that counseling records and mental health information are kept separate from the teacher organization’s human resources and supervisory systems, thereby enhancing confidentiality and trust. These features help reduce concerns about mental illness, organizational stigma, and potential adverse consequences, thereby encouraging teachers to seek mental health services when needed [43–47].

### 2. Health and Safety Executive Management Standards for managing work-related stress and organization-based mental health management systems in the United Kingdom

The United Kingdom’s school behavior management system uses a relatively structured approach to behavioral interventions and student support to protect teachers’ educational activities and students’ rights to learn. Students with severe behavioral problems may be temporarily removed from the general classroom to receive education and behavioral interventions in a separate behavioral support space, with school administrators and behavioral support staff sharing the responsibility for this process [48]. Furthermore, some high-risk students may be referred to alternative educational institutions, such as pupil referral units, with the support of local authorities, where the students receive educational, psychological, and behavioral support [49]. This system emphasizes multidisciplinary collaboration among teachers, psychological specialists, counselors, and social and health care professionals and includes a structured approach designed to prevent responsibility for supporting students with behavioral problems from being unduly concentrated on individual teachers [50].

The United Kingdom is distinctive in approaching mental health issues among school staff, not as individual vulnerabilities, but as occupational safety and health issues to be managed by organizations. The United Kingdom Health and Safety Executive (HSE) defines work-related stress as a significant occupational risk factor through its management standards and requires employers to conduct risk assessments to prevent harm to employees’ mental and physical health [51,52].

The HSE Management Standards organize the key risk areas related to work-related stress into six categories: demands, control, support, relationships, role, and change. These include excessive workload and time pressure, low job autonomy, lack of support from managers and colleagues, conflict and harassment, role ambiguity, and uncertainty during organizational change [51]. This framework is significant because it encourages viewing job stress not merely as a matter of individual competence or adaptation but as a challenge that can be addressed at the organizational level.

In its mental health guidelines for school organizations, the United Kingdom Education Support agency recommends conducting stress risk assessments, providing training for administrators, coordinating with occupational health specialists, using counseling and EAPs, and implementing programs to improve mental health awareness [53]. This approach can be understood as a preventive occupational health model that supports the assessment and management of job stressors, such as staff workload, administrative burdens, conflicts with parents and students, organizational support, and role clarity, at the organizational level [1].

### 3. Finland’s multidisciplinary collaboration model based on the student welfare team

Finland takes a preventive and multidisciplinary approach to students’ emotional and behavioral issues through a legally established student welfare system under the “Student Welfare Act” [54,55]. In schools, this system is operated by a multidisciplinary student welfare team comprising the principal or vice principal, homeroom teachers, school psychologists, school social workers, and school nurses; the school physician is also included when necessary [56]. This approach ensures that all students have the right to receive appropriate welfare services when needed [57]. The team identifies learning difficulties, emotional and behavioral problems, mental health risk factors, and difficulties adapting to school at an early stage and discusses strategies for intervention. With the consent of the students and their guardians, the team conducts psychosocial assessments and provides counseling to the guardians. In addition, the team develops school-based support plans and, when necessary, coordinates with external health and social welfare services. In this process, teachers play a crucial role in identifying student difficulties early, and therapeutic interventions and mental health management are primarily conducted collaboratively through the multidisciplinary student welfare system. This division of responsibilities serves as an environmental safeguard. This reduces the burden and role overload that teachers face when handling student mental health issues alone, helping them focus more on their core role as educators [10].

Finland’s student well-being model emphasizes prevention and early intervention rather than reacting after problems have arisen [58]. This approach aligns with public mental health and “whole school” approaches aimed at protecting students’ mental health and maintaining the psychological safety of teachers and the broader school community [45,59]. This multidisciplinary student welfare system can serve as an organizational buffer to alleviate teachers’ emotional burden and secondary post-traumatic stress [18].

Since educational governance, occupational health systems, and mental health service delivery differ across countries, these models cannot be directly applied to South Korea. Their relevance lies in the organizational principles they illustrate, including early identification, confidential access to professional support, organizational responsibility for occupational stress, multidisciplinary management of complex student needs, and structured referral pathways to mental health services. These principles should be adapted to the Korean educational context, considering existing institutional roles, regional resource disparities, confidentiality concerns, and the availability of external mental health services.

## Weaknesses in the current mental health support system for teachers and limitations of crisis intervention

In August 2023, the Ministry of Education announced the “*Comprehensive plan to restore and strengthen the protection of teachers’ rights*” [31]. These policy initiatives are significant because they recognize teachers’ mental health issues as public mental health concerns that are linked to the sustainability of the entire public education system. However, despite these institutional efforts, challenges, such as the difficulty teachers face in accessing mental health support services in actual school settings and the lack of a systematic and professional support system for teachers exposed to crises or trauma, remain areas that require improvement [53,60].

### 1. Structural stigma and barriers to help-seeking

Through its plan to support teachers’ mental health recovery, the government provides psychological assessments, counseling, referrals to private-sector experts, and support for psychiatric treatment. Institutional accessibility is also expanding. However, despite the existence of mental health support systems, service utilization is limited in practice [61]. This limited utilization may be due to the structural stigma and self-stigma surrounding mental illness and the use of counseling services [62]. Concerns that disclosing mental health issues could negatively affect professional reputation or career prospects have been reported as major barriers to seeking help across various occupational groups [46,47].

Furthermore, there are structural challenges that make it difficult for individual teachers to find time for counseling and treatment because of student guidance and administrative duties in the actual school environment. Heavy workloads can serve as a practical barrier to accessing mental health services, and as emotional exhaustion and depressive symptoms worsen, teachers may be less likely to seek help [63,64]. These factors can delay the use of mental health services by teachers, which in turn can lead to delays in the early detection of mental health problems and timing of therapeutic interventions [65].

If teachers repeatedly experience situations in which they perceive inadequate protection from society, school districts, or school organizations, they may endure and cope on their own rather than actively disclosing problematic situations or seeking help [66]. According to a 2025 study on the harm experienced by teachers during educational activities and proposals for legal and policy improvements, 17.8% of teachers who had experienced such harm reported not disclosing the incident to anyone [67]. The most common reason, accounting for 50.2% of responses, was that teachers did not believe that speaking up would lead to a resolution. Furthermore, 65.2% of those who reported harm responded that no action had been taken. Therefore, future mental health support systems for teachers need to move beyond simply expanding service provision and to focus on strengthening psychological safety and trust in the confidentiality of mental health services. In particular, counseling and treatment information should be managed independently of personnel evaluation systems, and more teacher-friendly mental health support pathways should be established to ensure that teachers at high risk do not delay seeking treatment.

### 2. Lack of trauma-focused crisis intervention tailored to teachers

Currently, South Korea’s public education system has no standardized emergency psychological stabilization programs or professional support services for school staff that can be immediately activated on campus in the event of a traumatic incident. The school crisis intervention system was primarily designed to protect students and restore school operations. While this system includes mental health support for school staff, key procedures and specific intervention strategies are lacking, and the focus is predominantly on students [68]. Furthermore, trauma-informed psychological interventions that adequately address emotional distress experienced by teachers are lacking [69].

Teachers who have experienced traumatic events, such as disruptions to educational activities, conflicts related to reporting incidents, verbal abuse or physical assault, or student suicide, require a standardized crisis intervention system that goes beyond simple counseling. Such a system should ensure immediate safety after an incident, provide psychological first aid, conduct ongoing monitoring, and refer individuals to specialized treatment facilities [70]. Moving forward, the mental health support system for teachers must go beyond post-incident counseling to establish a rapid and accessible clinical support system starting from the early stages of a traumatic event [71].

## Recommendations for a Korean-style teacher employee assistance program model

Based on the mechanisms underlying the mental health crises of teachers discussed above and the international models of educator support, this review proposes the following core components of a Korean-style EAP model to protect and restore teachers’ mental health (Fig. 2).

### 1. Employee assistance program model for protecting teachers’ mental health

To establish an effective system to protect teachers’ mental health, it is necessary to consider an externally contracted EAP model that operates independently of the organization [72]. Research on occupational mental health and occupational health has consistently indicated that concerns about confidentiality and fear of adverse personnel actions are major barriers that delay help-seeking [73]. Particularly in professions such as teaching, where organizational reputation and evaluation systems have a significant influence, structural and self-stigma are likely to hinder the use of mental health services [46].

An EAP operated by an external, independent organization can enhance trust in confidentiality and independence, thereby promoting the use of mental health services [74]. Therefore, the mental health support system for school staff must also have an independent operational structure that separates counseling requests, clinical record management, mental health data storage, and referrals to specialized treatment from educational administration and human resource systems. Such an independent EAP model has the potential to function as a public mental health system that goes beyond post-incident counseling by connecting faculty and staff at high risk for mental health issues to appropriate support systems at an early stage and by promoting preventive mental health interventions.

### 2. Strengthening the crisis response and mental health protection system led by school administrators

The school EAP system should not be limited to counseling individual frontline teachers; rather, it should be expanded to provide systematic consultation and crisis-response support to school administrators, such as principals and vice principals. School administrators can serve as important gatekeepers by recognizing early warning signs of psychological crises among faculty and staff, connecting them to appropriate support systems, and ensuring that affected teachers do not experience further secondary harm from inappropriate organizational responses [69].

When major incidents occur, such as student suicide, severe parental violence, or high-intensity conflicts arising from public complaints, school administrators should consider using standardized crisis-response protocols rather than relying solely on personal judgment [75]. They should also activate a coordination system linking the relevant city or provincial education office, EAPs, local psychiatric departments, and mental health welfare centers. Such a system can ensure immediate safety after an incident, protect affected school staff, provide psychological first aid, screen high-risk groups, and refer individuals to specialized mental health services.

Accordingly, city and provincial education offices should consider incorporating mental health gatekeeper training and crisis response drills for principals and vice principals into their regular administrative training programs [76]. A systematic review and meta-analysis of workplace mental health training for administrators indicated that such training significantly improved administrators’ knowledge, attitudes, and support behaviors regarding mental health [77].

In the long term, efforts to enhance staff psychological safety and protect mental health should be incorporated into school leadership evaluations and administrator competency development programs to support a trauma-informed organizational culture.

### 3. Establishing an organizational framework for preventive mental health interventions

From the perspective of preventive occupational mental health, establishing a system for regular mental health risk assessments is important for early identification of mental health risk factors in the teaching profession and for monitoring hazardous environments at the organizational level. In the teaching profession, the Korean Occupational Stress Scale [78] can be used to assess job-related psychosocial stress, whereas the Korean version of the Depression, Anxiety and Stress Scale-21 [79] can be used to screen for symptoms of depression, anxiety, and stress. In addition, the recently developed Korean Post-Traumatic Stress Scale for Teachers can be used to assess trauma responses specific to the teaching profession, such as those related to malicious complaints, interference with educational activities, and repeated emotional exhaustion [80].

Consistently high mental health risk indicators in a specific school or region can be interpreted as organizational-level warning signs reflecting not only the vulnerability of individual teachers but also an imbalance between job demands and job resources [81]. Therefore, mental health risk assessments should not be limited to simple screening but should lead to organizational-level interventions aimed at improving structural risk factors for job stress, such as workload, role clarity, organizational support systems, and conflict management systems.

### 4. Establishing a trauma-informed crisis intervention and specialized treatment referral system

Domestic studies have also indicated that teachers may experience post-traumatic stress reactions related to disruptions in educational activities, repeated complaints, and exposure to student crisis incidents [80]. Therefore, school EAPs must function as systematic critical incident response systems beyond counseling. Such crisis-response systems may include ensuring physical safety immediately after an incident, temporary removal from high-risk environments, consultation with administrators, psychological first aid, screening for trauma reactions, and, when necessary, prompt referral to psychiatric services and evidence-based trauma treatment [82]. Repeated violations of educational activities, exposure to high-intensity complaints, and experiences of institutional betrayal can go beyond general job stress, leading to moral injury, embitterment, and distrust of the organization. Therefore, trauma-informed assessment and linkage to specialized mental health treatment are necessary [13,22,26,71].

Accordingly, education offices and independent EAP systems must strengthen rapid referral networks linked to local psychiatrists, mental health welfare centers, and crisis intervention teams to ensure that high-risk school staff can access appropriate mental health services without delay. During the early intervention stage, a preventive occupational mental health system should be developed to minimize financial and administrative barriers to treatment.

## Conclusion

This review reexamined the mental health issues that have recently emerged in the Korean teaching profession through concepts such as moral injury, institutional betrayal, secondary traumatic stress, and psychological safety, and proposes a new theoretical framework for understanding these issues. It focused on the possibility that teachers who enter the profession with high moral expectations and a strong professional identity may experience damage to their professional identity and value systems, going beyond simple emotional burnout, as they are exposed to rapid changes in the educational environment and repeated psychosocial stress.

We emphasized that teachers’ mental health issues may not stem solely from a lack of individual resilience or failure to adapt but may result from accumulated experiences of repeated moral conflict, organizational stress, and institutional betrayal. Therefore, structural approaches to mitigate chronically threatening environments and organizational risk factors must be implemented in parallel.

The international cases reviewed in this paper suggest that protecting teachers’ mental health should be viewed not merely as a provision of welfare services but rather as a preventive occupational mental health strategy aimed at promoting psychological safety and restoring organizational trust. EAPs that ensure independence and confidentiality, organization-based mental health risk assessments, multidisciplinary support systems, and trauma-informed organizational frameworks can serve as key components in protecting the mental health of teachers.

## Figures and Tables

**Fig. 1.**
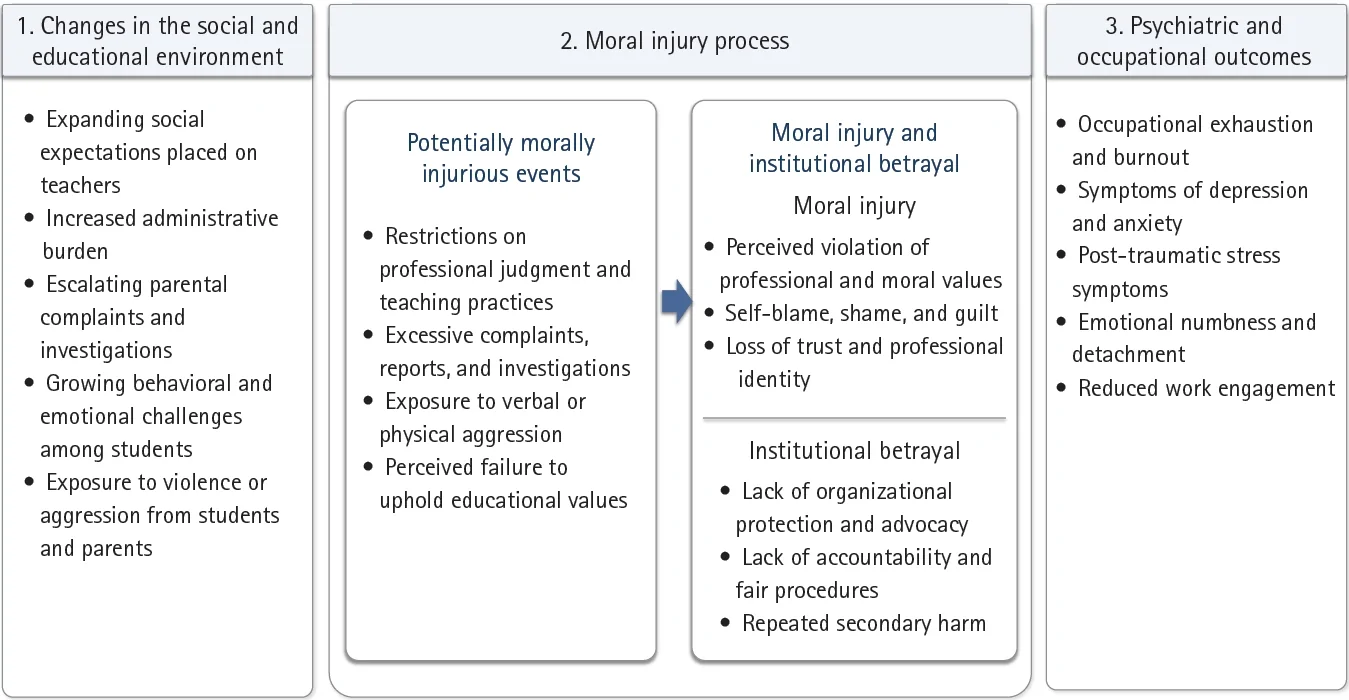
Moral injury and institutional betrayal in teachers’ mental health.

**Fig. 2.**
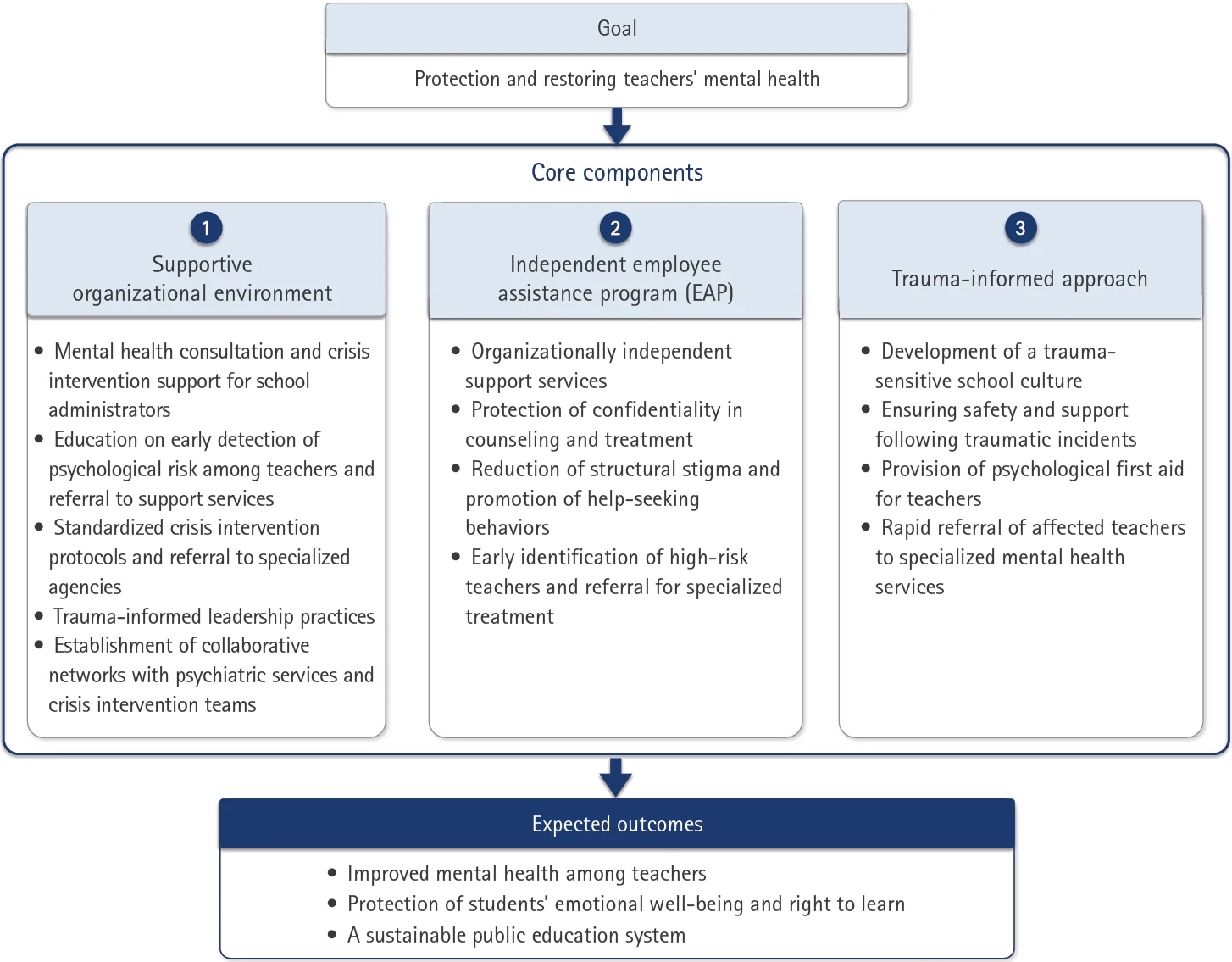
Proposed Korean teacher employee assistance program (EAP) model for mental health protection.

**Table 1.** International organizational models relevant to teacher mental health and their implications for South Korea

Country	System or model	Primary target	Main approach	Potential relevance to Korea	Limitation in application
United States	Multitiered system of supports/Positive Behavioral Interventions and Supports; external EAP	Students; school staff	Tiered multidisciplinary student support; confidential external EAP	May inform multidisciplinary support for high-risk students and confidential teacher EAP services	Requires adaptation to differences in specialized staffing and EAP infrastructure
United Kingdom	Structured behavior support; Health and Safety Executive Management Standards	Students; school staff/organizations	Structured behavior support and organization-level management of work-related stress	May inform psychosocial risk assessment, administrator responsibility, and organizational stress management	Differences in occupational health systems, employer responsibilities, and school support infrastructure
Finland	Multidisciplinary student welfare system	Primarily students	Preventive, school-based multidisciplinary support and early intervention	May inform multidisciplinary collaboration and shared responsibility for students with complex needs	Requires adequate multidisciplinary resources; not a teacher-specific EAP

EAP, employee assistance program.
